# Combination of alpha-fetoprotein and neutrophil-to-lymphocyte ratio to predict treatment response and survival outcomes of patients with unresectable hepatocellular carcinoma treated with immune checkpoint inhibitors

**DOI:** 10.1186/s12885-023-11003-0

**Published:** 2023-06-15

**Authors:** Hong-Fei Zhu, Jin-Kai Feng, Yan-Jun Xiang, Kang Wang, Li-Ping Zhou, Zong-Han Liu, Yu-Qiang Cheng, Jie Shi, Wei-Xing Guo, Shu-Qun Cheng

**Affiliations:** 1 Department of Hepatic Surgery VI, Eastern Hepatobiliary Surgery Hospital, Naval Medical University, Shanghai, China; 2 Department of Hepatobiliary Surgery, The First Affiliated Hospital of Wenzhou Medical University, Zhejiang, China; 3 Eastern Hepatobiliary Surgery Hospital, Naval Medical University, 225 Changhai Road, Shanghai, 200433 China

**Keywords:** Hepatocellular carcinoma (HCC), Immune checkpoint inhibitor (ICI), Immunotherapy, Predictive score, Overall survival (OS), Progression-free survival (PFS), Alpha-fetoprotein (AFP), Neutrophil-to-lymphocyte ratio (NLR)

## Abstract

**Background:**

Immune-checkpoint inhibitors (ICIs) have revolutionized the treatment of hepatocellular carcinoma (HCC). However, long-term survival outcomes and treatment response of HCC patients undergoing immunotherapy is unpredictable. The study aimed to evaluate the role of alpha-fetoprotein (AFP) combined with neutrophil-to-lymphocyte ratio (NLR) to predict the prognosis and treatment response of HCC patients receiving ICIs.

**Methods:**

Patients with unresectable HCC who received ICI treatment were included. The HCC immunotherapy score was developed from a retrospective cohort at the Eastern Hepatobiliary Surgery Hospital to form the training cohort. The clinical variables independently associated with overall survival (OS) were identified using univariate and multivariate Cox regression analysis. Based on multivariate analysis of OS, a predictive score based on AFP and NLR was constructed, and patients were stratified into three risk groups according to this score. The clinical utility of this score to predict progression-free survival (PFS) and differentiate objective response rate (ORR) and disease control rate (DCR) was also performed. This score was validated in an independent external validation cohort at the First Affiliated Hospital of Wenzhou Medical University.

**Results:**

Baseline AFP ≤ 400 ng/ml (hazard ratio [HR] 0.48; 95% CI, 0.24–0.97; *P* = 0.039) and NLR ≤ 2.77 (HR 0.11; 95% CI, 0.03–0.37; *P*<0.001) were found to be independent risk factors of OS. The two labolatory values were used to develop the score to predict survival outcomes and treatment response in HCC patients receiving immunotherapy, which assigned 1 point for AFP > 400 ng/ml and 3 points for NLR > 2.77. Patients with 0 point were classified as the low-risk group. Patients with 1–3 points were categorized as the intermediate-risk group. Patients with 4 points were classified as the high-risk group. In the training cohort, the median OS of the low-risk group was not reached. The median OS of the intermediate-risk group and high-risk group were 29.0 (95% CI 20.8–37.3) months and 16.0 (95% CI 10.8–21.2) months, respectively (*P* < 0.001). The median PFS of the low-risk group was not reached. The median PFS of the intermediate-risk group and high-risk group were 14.6 (95% CI 11.3–17.8) months and 7.6 (95% CI 3.6–11.7) months, respectively (*P* < 0.001). The ORR and DCR were highest in the low-risk group, followed by the intermediate-risk group and the high-risk group (*P* < 0.001, *P* = 0.007, respectively). This score also had good predictive power using the validation cohort.

**Conclusion:**

The HCC immunotherapy score based on AFP and NLR can predict survival outcomes and treatment response in patients receiving ICI treatments, suggesting that this score could serve as a useful tool for identification of HCC patients likely to benefit from immunotherapy.

**Supplementary Information:**

The online version contains supplementary material available at 10.1186/s12885-023-11003-0.

## Highlights

Baseline serum AFP > 400 ng/mL and NLR > 2.77 were independent prognostic factors associated with worse OS and PFS in unresectable HCC patients treated with immune checkpoint inhibitors.

A scoring system based on these two variables predicts survival outcomes and treatment response in patients with unresectable HCC undergoing immunotherapy.

**Supplementary Information:**

The online version contains supplementary material available at 10.1186/s12885-023-11003-0.

## Introduction

Hepatocellular carcinoma (HCC) is the sixth most common cancer and ranks as the fourth leading cause of cancer-related death worldwide [1]. Although HCC patients with early-stage disease can be radically cured by liver transplantation or resection, most patients fail to meet the surgical criteria at the time of diagnosis due to tumor burden or underlying cirrhosis, thus having a poor prognosis [2].

With rapid development of immunotherapy, the treatment landscape for malignancies has dramatically changed over the past few years. The safety and efficacy of immune checkpoint inhibitors (ICIs) have been extensively investigated in patients with HCC. The combination regimen of atezolizumab with bevacizumab is now regarded as the new reference standard in first-line systemic treatment for unresectable HCC [3]. Pembrolizumab, nivolumab, and the combination of nivolumab plus ipilimumab are now proposed as second-line treatment options by the United States FDA [4, 5].

In China, ORIENT-32 trial demonstrated a positive result that sintilimab plus a bevacizumab biosimilar was superior to sorafenib alone for unresectable or metastatic HCC [6]. Camrelizumab and tislelizumab are now considered second-line treatment options for advanced HCC patients who are initially first-line treatment failure [7, 8]. However, the treatment efficacy and response rates of ICIs vary greatly among HCC patients, and only a small proportion of patients can benefit from immunotherapy [7]. Currently, several predictive biomarkers for immunotherapy have been identified, including programmed death ligand 1 (PD-L1) expression and activated Wnt/β-catenin signalling pathway [9–11]. Nevertheless, these markers can only be measured on the basis of acquirement of tumor tissues, and the utility is limited since invasive procedures should be performed. Therefore, in such circumstances, building a practical and reliable scoring system based on serological noninvasive biomarkers to guide treatment decision-making and predict the survival outcomes of ICI therapy for unresectable HCC is urgently needed and desirable in clinical practice.

Several retrospective studies demonstrated that baseline serum alpha-fetoprotein (AFP) level and early on-treatment response of AFP were associated with therapeutic efficacy and prognosis for HCC patients treated with ICI-based regimens [12–14]. Moreover, a meta-analysis showed that elevated pretreatment blood neutrophil-to-lymphocyte ratio (NLR) was a promising prognostic biomarker for advanced-stage cancer patients treated with immunotherapy [15]. These suggested that the possibility of combining tumor and inflammatory biomarkers to assist in the identification of HCC patients who benefit from immunotherapy.

In the present study, we developed a simple and easily applicable scoring system based on serological AFP and NLR to predict treatment response and survival outcomes in patients with unresectable HCC undergoing immunotherapy with ICIs.

## Methods and materials

### Ethical statement

This retrospective study was conducted in accordane with the International Conference on Good Clinical Practice Standards and the ethical guidelines of Declaration of Helsinki. Documented approval was obtained from the Clinical Research Ethics Committee of the Eastern Hepatobiliary Surgery Hospital (EHBH) and the First Affiliated Hospital of Wenzhou Medical University. Written informed consent was obtained from all the patients for research purposes. Patients’ details have been anonymized to protect the privacy of patients.

### Study design and patients

Patients with unresectable HCC who underwent anti-programmed death (ligand) 1 (PD-(L)1)-based immunotherapy were considered for this study. HCC was diagnosed radiologically or pathologically based on the American Association for the Study of Liver Diseases (AASLD) practice guidelines [16]. Immunotherapy was initiated between March 2019 and June 2021 at EHBH. Patients who received immunotherapy in combination with tyrosine kinase inhibitors (TKIs) or locoregional therapies (LRTs) were also included in this study. Patients who received immunotherapy as adjuvant treatment after curative therapies were excluded. This group formed the internal training cohort of this study. The patients with unresectable HCC who received ICIs from the First Affiliated Hospital of Wenzhou Medical University at the same time period were collected as the external validation cohort.

Clinical data including patients’ demographics, disease background, imaging information, laboratory results, and treatment regimens were retrospectively collected from electronic medical records of two participating hospitals. The start of ICBs therapy was considered the baseline. All laboratory data were obtained within three days before the initiation of immunotherapy. Radiological imaging assessment was performed based on contrast-enhanced computed tomography (CT) or magnetic resonance imaging (MRI) within one week before the initial treatment.

### Eligibility criteria

The inclusion criteria were [1] age ≥ 18 years; [2] unresectable HCC that was classified as Barcelona Clinic Liver Cancer (BCLC) stage B or C; [3] treatment-naive; [4] well-preserved liver function of Child-Pugh class A or B7; [5] had an Eastern Cooperative Oncology Group (ECOG) performance status of 0–2; [6] had at least one measurable lesion as defined by the modified Response Evaluation Criteria in Solid Tumors (mRECIST) [17]; [7] had adequate hematologic and organ function; and [8] had a predicted life expectancy of more than 12 weeks. The exclusion criteria were [1] recurrent liver cancer; [2] a history of other malignancies; [3] had contraindications for systemic therapy; [4] baseline serum AFP, neutrophil, or lymphocyte values were not available; and [5] patients who were lost to follow-up. The same inclusion and exclusion criteria were used in the validation cohort.

### PD-(L)1-based immunotherapy

After a comprehensive discussion of all cases at weekly multidisciplinary treatment (MDT) meetings including liver surgeons, hepatologists, interventional radiologists, and medical oncologists, the patients were recommended to be treated with anti-PD-(L)1 antibodies-based therapies. Patients were fully informed of the treatment effectiveness, potential adverse events (AEs), and medical costs of immunotherapy.

In this study, four types of PD-1 inhibitors (Camrelizumab, Sintilimab, Toripalimab, Tislelizumab) and one type of PD-L1 inhibitor (Atezolizumab) were administered intravenously at the standard dose every 3 weeks according to the instructions of pharmaceutical companies (**Table **S1). When minor immune-related complications or infusion reactions occurred, dose reduction or treatment interruption was permitted. Immunotherapy was immediately discontinued after any intolerable severe toxicities, tumor progression, or patient withdrawal of consent to participate.

Loco-regional therapies (LRTs) such as transarterial chemoembolization (TACE) and ablation were performed before immunotherapy. Tyrosine kinase inhibitors (TKIs), such as sorafenib and lenvatinib, were prescribed synchronously or sequentially with anti-PD-(L)1 drugs.

### Follow-up, assessment and study endpoints

All patients were regularly followed up at the outpatient clinic of the two hospitals. At each follow-up visit, physical examination, laboratory test, abdominal ultrasound, enhanced CT and/or MRI were routinely performed. Radiological response of tumors was evaluated independently by two professional radiologists at baseline and every 6–12 weeks thereafter. The primary endpoint of this study was overall survival (OS), which was defined as the time from initiation of immunotherapy until death, or patients who were still alive when censored at the date of last contact. The secondary endpoints were progression-free survival (PFS), objective response rate (ORR), and disease control rate (DCR). PFS referred to the time from the start of ICBs treatment to the first radiologically confirmed tumor progression, death, or last contact. ORR and DCR were assessed in accordance with the HCC-specific modified Response Evaluation Criteria in Solid Tumors (mRECIST). ORR was calculated as the sum of complete response (CR) and partial response (PR). DCR was defined as the sum of CR, PR, and stable disease (SD). This study was censored on September 1st, 2021.

### Statistical analysis

As this is a retrospective study, no formal sample size estimation was performed, instead, all patients fulfilling the inclusion and exclusion criteria were considered eligible for this study.

Descriptive statistics were used to summarize the data on baseline clinical characteristics. Continuous data were presented as mean with standard deviation or median with interquartile range, and compared using the Student’s *t* test or the Mann-Whitney *U* test as appropriate. Categorical variables were expressed as numbers and percentages, and compared using the Chi-square test or Fisher’s exact test. Survival curves were generated by Kaplan-Meier method and compared by the means of log-rank test. The median estimated follow-up time was calculated using the reverse Kaplan-Meier method [18].

Univariable and multivariable analyses were performed using Cox regression models to determine the independent prognostic factors for OS and PFS. Variables with a *P* value less than 0.05 on univariable analysis were incorporated into multivariable analysis. The regression coefficients (β) of the Cox regression model were divided by the median of the regression coefficients (β) of all the parameters in the model and approximated to the nearest unit (1.00 units) to obtain simple point numbers to facilitate calculation of the immunotherapy score. The receiver-operating characteristic (ROC) curve of NLR was performed to determine the optimal cutoff value to discriminate survival. To avoid overoptimistic evaluation of the model using the same data set, the treatment response estimation and prognostic performance of the scoring system were assessed in an independent external validation cohort from the First Affiliated Hospital of Wenzhou Medical University.

All the reported *P* values were two-sided. Statistical significance was set at *P* < 0.05 in this study. IBM SPSS Statistics (version 24.0, SPSS Inc., Chicago, IL), R program (version 4.0.2, R foundation for Statistical Computing, Vienna, Austria) and MedCalc (version 20.027, MedCalc Software Ltd., Ostend, Belgium) were used to perform statistical analyses and visualize the results.

## Results

### Baseline characteristics of patients

**Fig. 1:**
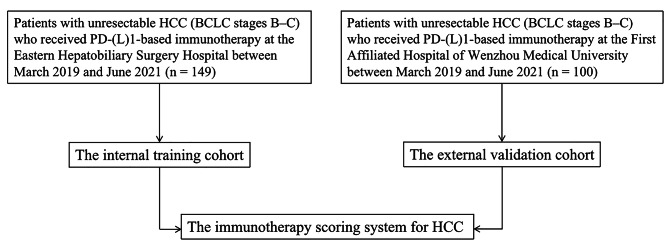
Flow chart showing the selection process of patients with unresectable HCC at BCLC stage B or C who received PD-(L)1-based immunotherapy in the training cohort (n = 149) and the external validation cohort (n = 100) Abbreviations: HCC, hepatocellular carcinoma; BCLC, Barcelona Clinic Liver Cancer; PD-(L)1, programmed death (ligand) 1

As shown in Fig. 1, One-hundred and forty-nine patients (125 male and 24 female) receiving anti-PD-(L)1-based immunotherapy met the eligibility criteria in the training cohort. One-hundred patients (79 male and 21 female) undergoing anti-PD-(L)1-based immunotherapy formed the validation cohort. The baseline characteristics of the training and validation cohorts are described in Table 1, and category and dosage of immunotherapeutic agents used are shown in **Table **S1.

**Table 1:** Baseline patient demographics and disease characteristics of the internal training and external validation cohorts of patients

Characteristics	Internal training cohort N = 149 (%)	External validation cohort N = 100 (%)
Age, years	56 (48–61)	56 (47–62)
Age, years		
<65	123 (82.6)	86 (86.0)
≥ 65	26 (17.4)	14 (14.0)
Sex		
Male	125 (83.9)	79 (79.0)
Female	24 (16.1)	21 (21.0)
Etiology		
HBV	125 (83.9)	86 (86.0)
Non-HBV	24 (16.1)	14 (14.0)
Cardiovascular diseases		
Presence	95 (63.8)	59 (59.0)
Absence	54 (36.2)	41 (41.0)
T2DM		
Presence	42 (28.2)	25 (25.0)
Absence	107 (71.8)	75 (75.0)
Antiviral treatment		
Yes	72 (48.3)	46 (46.0)
No	77 (51.7)	54 (54.0)
Child-Pugh class		
A	129 (86.6)	84 (84.0)
B	20 (13.4)	16 (16.0)
ECOG PS score		
0–1	141 (94.6)	98 (98.0)
2	8 (5.4)	2 (2.0)
BCLC stage		
B	104 (69.8)	70 (70.0)
C	45 (30.2)	30 (30.0)
PLT, ×10^9^/L	157.6 ± 59.1	157.8 ± 59.7
PT, s	12.3 (11.7–13.1)	12.4 (11.9–13.2)
Scr, umol/L	67 (58–80)	62 (56–67)
Blood glucose, mmol/L	5.18 (4.60–6.24)	5.20 (4.64–6.30)
TBIL, umol/L	16.9 (12.4–22.7)	16.0 (12.1–22.7)
ALB, g/L	39.6 (35.7–42.4)	39.5 (35.4–42.1)
ALBI score	-2.52 (-2.85–-2.11)	-2.50 (-2.83–-2.08)
ALBI grade		
I	67 (45.0)	42 (42.0)
II	78 (52.3)	54 (54.0)
III	4 (2.7)	4 (4.0)
AFP, ng/ml		
≤ 400	76 (51.0)	52 (52.0)
>400	73 (49.0)	48 (48.0)
NLR	2.98 (2.13–4.32)	2.94 (2.05–4.31)
NLR		
≤ 2.77	62 (41.6)	45 (45.0)
> 2.77	87 (58.4)	55 (55.0)
DCP, mAU/ml		
≤ 400	47 (31.5)	34 (34.0)
> 400	102 (68.5)	66 (66.0)
HBV-DNA, copies/ml		
≤ 1000	124 (83.2)	84 (84.0)
> 1000	25 (16.8)	16 (16.0)
Macrovascular invasion		
Yes	45 (30.2)	30 (30.0)
No	104 (69.8)	70 (70.0)
Extrahepatic metastasis		
Yes	7 (4.7)	6 (6.0)
No	142 (95.3)	94 (94.0)
Tumor number		
Single	12 (8.1)	8 (8.0)
Multiple	137 (91.9)	92 (92.0)
Largest tumor size, cm	9.5 (6.8–13.0)	10.0 (6.7–13.2)
Combined treatment besides ICIs		
TACE	131 (87.9)	92 (92.0)
TKI*	90 (60.4)	65 (65.0)
PMCT	12 (8.1)	6 (6.0)
RT	3 (2.0)	4 (4.0)
Cycles of anti-PD-1/PD-L1 Median (range)	7 (2–25)	6 (3–18)

Notes: values are presented as mean ± SD, median (interquartile range), or numbers (percentages)

*TKI include Sorafenib, Lenvatinib, Regorafenib and Apatinib

HBV, hepatitis B virus; T2DM, type 2 diabetes mellitus; ECOG PS, Eastern Cooperative Oncology Group performance status; BCLC, Barcelona Clinic Liver Cancer; PLT, platelets; PT, prothrombin time; Scr, serum creatinine; TBIL, total bilirubin; ALB, albumin; ALBI, albumin-bilirubin; AFP, alpha-fetoprotein; NLR, neutrophil-to-lymphocyte ratio; DCP, des-γ-carboxy-prothrombin; ICIs, immune checkpoint inhibitors; TACE, transcatheter arterial chemoembolization; TKI, tyrosine kinase inhibitor; PMCT, percutaneous microwave coagulation therapy; RT, radiotherapy

### Optimal NLR cutoff to discriminate survival of patients

The optimal cutoff value of NLR to discriminate distinct survival in patients treated with immunotherapy was determined using the ROC curve. As shown in **Figure **S1, the optimal cutoff of NLR was 2.77, with the area under curve (AUC) of 0.759 (*P* < 0.001).

### OS and PFS of the internal training cohort

**Fig. 2:**
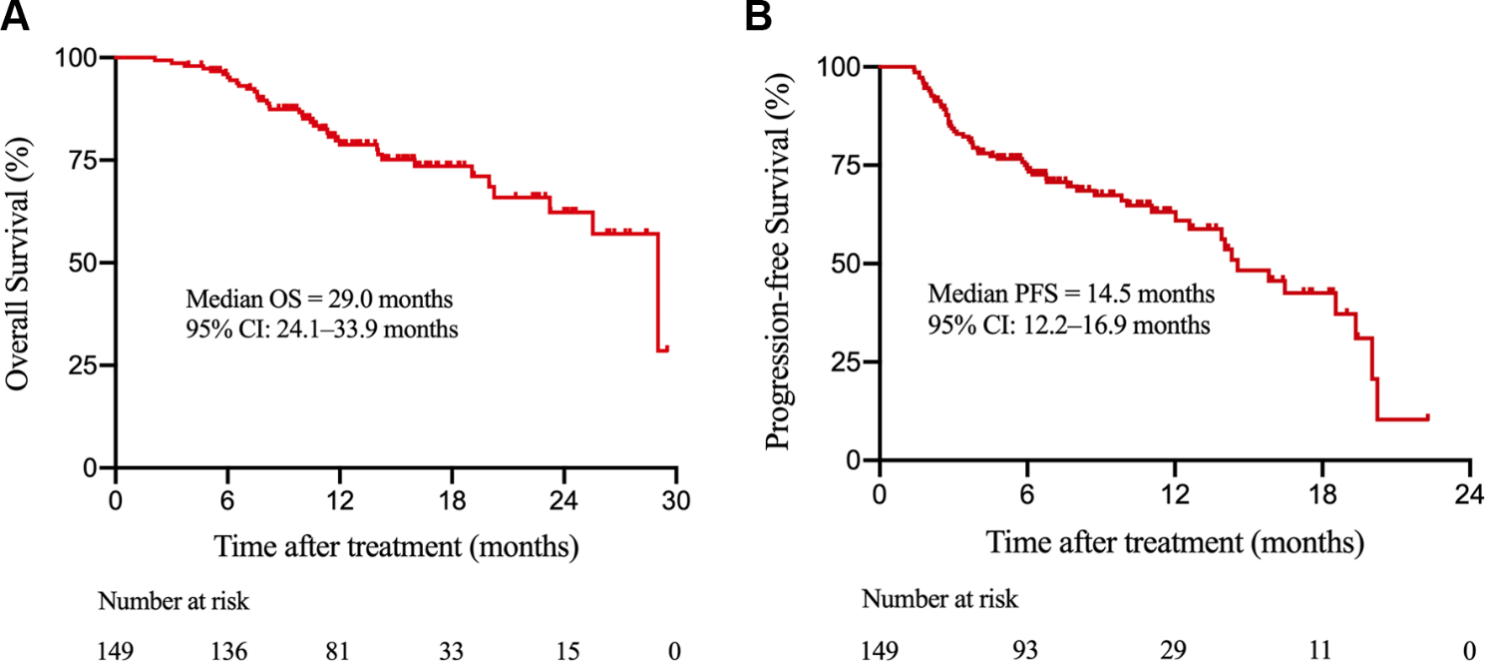
Kaplan-Meier survival curves of the internal training cohort of HCC patients undergoing immunotherapy. (**A**) overall survival (OS) curve of patients; (**B**) progression-free survival (PFS) curve of patients Abbreviations: OS, overall survival; PFS, progression-free survival

As of the data cutoff on September 1st, 2021, the median duration of estimated follow-up was 14.6 (95% CI 13.3–15.8) months. In the internal training cohort, the median OS was 29.0 (95% CI 24.1–33.9) months, and the median PFS was 14.5 (95% CI 12.2–16.9) months (Fig. 2).

### OS and PFS in the NLR and AFP subgroups of the internal training cohort

Some clinicopathological data, such as age, prothrombin time (PT), blood glucose, ALBI grade, and des-γ-carboxy-prothrombin (DCP), were markedly different between the low AFP (≤ 400 ng/mL) and high AFP (> 400 ng/mL) groups of the training and validation cohorts (**Table **S2). The percentages of patients who had HBV-DNA level > 1000 copies/ml were significantly higher for the NLR > 2.77 subgroup in both the training and validation cohorts. Other baseline clinical characteristics were not significantly different between the low NLR (≤ 2.77) and high NLR (> 2.77) groups of the training and validation cohorts (**Table **S3).

**Fig. 3:**
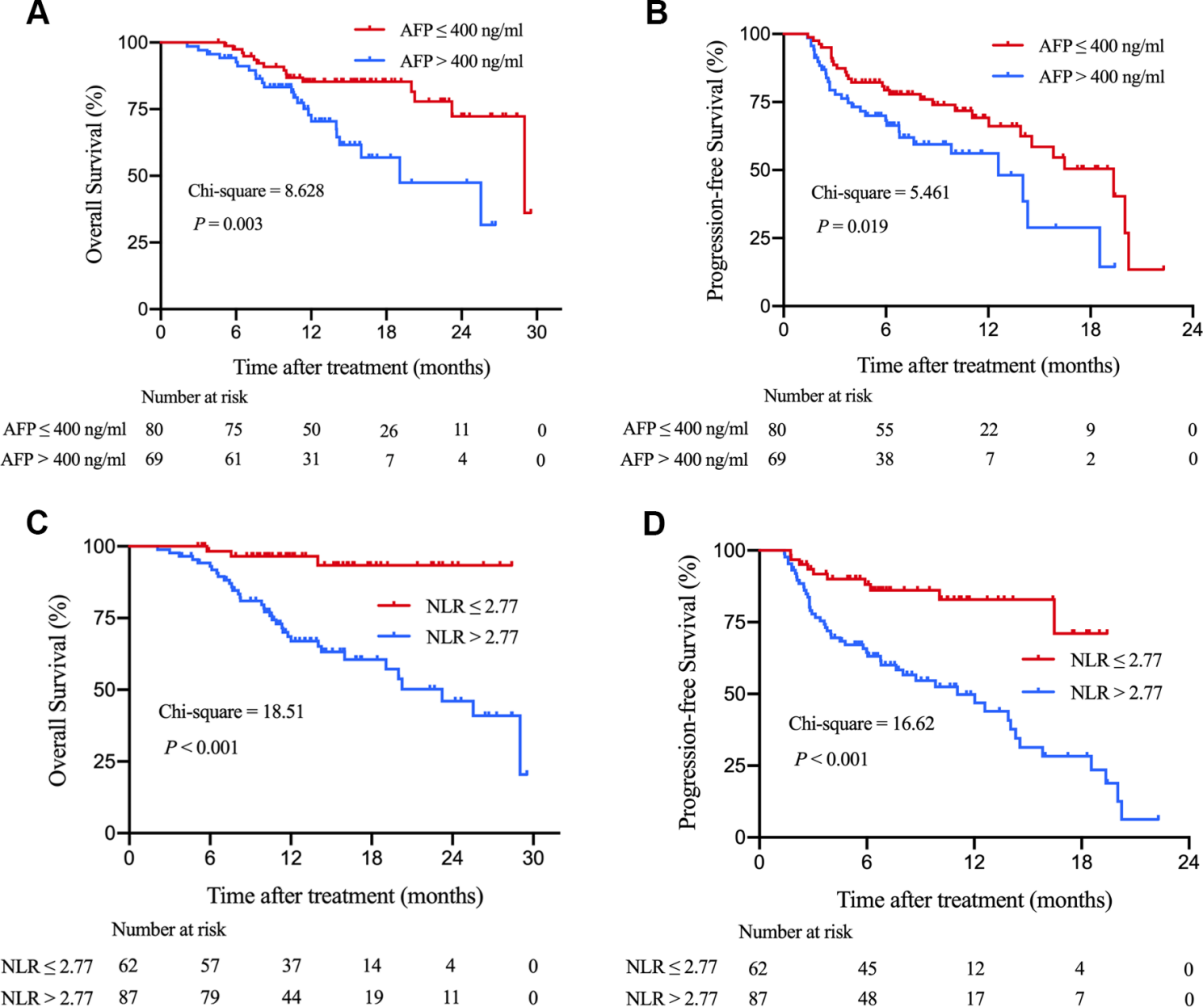
Kaplan-Meier survival curves of the internal training cohort of HCC patients undergoing immunotherapy stratified by AFP and NLR. (**A**) overall survival (OS) in the low and high AFP groups; (**B**) progression-free survival (PFS) in the low and high AFP groups; (**C**) OS in the low and high NLR groups; (**D**) PFS in the low and high NLR groups Abbreviations: AFP, alpha-fetoprotein; NLR, neutrophil-to-lymphocyte ratio

In the training cohort, the median OS and PFS were 29.0 (95% CI 20.9–37.1) and 19.4 (95% CI 13.5–25.2) months, respectively, for the low AFP group, compared with 19.1 (95% CI 11.0–27.2) and 12.6 (95% CI 8.2–17.0) months, respectively, for the high AFP group (for OS, *P* = 0.003, Fig. 3A; for PFS, *P* = 0.019, Fig. 3B). Median OS and PFS were both not reached for the low NLR group, compared with 23.2 (95% CI 17.1–29.3) and 11.1 (95% CI 6.7–15.5) months, respectively, for the high NLR group (for OS, *P* < 0.001, Fig. 3C; for PFS, *P* < 0.001, Fig. 3D).

### Univariate and multivariable cox regression analyses in the training cohort

In the training cohort, univarate analysis suggested that AFP > 400 ng/mL (*P* = 0.003), NLR > 2.77 (*P* < 0.001) and HBV-DNA > 1000 copies/ml (*P* = 0.004) were associated with worse OS. AFP > 400 ng/mL (*P* < 0.001), NLR > 2.77 (*P* < 0.001) and HBV-DNA > 1000 copies/ml (*P* = 0.001) were also associated with worse PFS. (Table 2).

**Table 2:** Univariable Cox regression analyses of factors associated with overall survival and progression-free survival

	Overall survival				Progression-free survival			
	β	SE	HR (95% CI)	*P* value	β	SE	HR (95% CI)	*P* value
Age, ≤ 65 vs. >65	-0.049	0.447	0.95 (0.40–2.29)	0.913	-0.208	0.338	0.81 (0.42–1.58)	0.538
Sex, male vs. female	0.697	0.603	2.01 (0.62–6.55)	0.248	0.345	0.435	1.41 (0.60–3.31)	0.427
Hepatitis virus infection, yes vs. no	0.011	0.405	1.01 (0.46–2.24)	0.979	0.221	0.342	1.25 (0.64–2.44)	0.518
Cardiovascular diseases, yes vs. no	0.260	0.298	1.30 (0.72–2.32)	0.383	0.079	0.353	1.09 (0.55–2.18)	0.823
T2DM, yes vs. no	0.663	0.446	1.94 (0.81–4.65)	0.138	0.012	0.302	1.02 (0.56–1.83)	0.967
Antiviral treatment, yes vs. no	-0.531	0.345	0.59 (0.30–1.16)	0.124	-0.225	0.265	0.80 (0.48–1.34)	0.394
Child-Pugh stage, A vs. B	-0.219	0.483	0.80 (0.31–2.07)	0.651	-0.213	0.334	0.81 (0.42–1.56)	0.524
ECOG PS, 2 vs. 0–1	0.244	0.731	1.28 (0.31–5.35)	0.738	0.242	0.596	1.27 (0.40–4.10)	0.685
PLT, ≤ 100 vs. >100, ×10^9^/L	0.090	0.489	1.09 (0.42–2.86)	0.854	-0.091	0.410	0.91 (0.41–2.04)	0.825
PT, > 14 vs. ≤14, s	0.680	0.730	1.98 (0.47–8.25)	0.351	0.193	0.471	1.21 (0.48–3.06)	0.682
Scr, ≤ 84 vs. >84, umol/L	-0.299	0.423	0.74 (0.32–1.70)	0.480	-0.151	0.371	0.86 (0.42–1.78)	0.685
Blood glucose, ≤ 6.1 vs. >6.1, mmol/L	-0.107	0.371	0.90 (0.43–1.86)	0.774	-0.252	0.302	0.78 (0.43–1.40)	0.403
TBIL, > 17.1 vs. ≤17.1, umol/L	0.562	0.340	1.76 (0.90–3.42)	0.098	0.212	0.266	1.24 (0.73–2.08)	0.426
ALB, > 35 vs. ≤35, g/L	-0.582	0.373	0.56 (0.27–1.16)	0.118	-0.157	0.322	0.85 (0.45–1.61)	0.626
AFP, ≤ 400 vs. >400, ng/mL	-1.168	0.390	0.31 (0.15–0.67)	**0.003**	-1.204	0.327	0.30 (0.16–0.57)	**< 0.001**
NLR, ≤ 2.77 vs. >2.77	-2.154	0.603	0.12 (0.04–0.38)	**< 0.001**	-1.325	0.349	0.27 (0.13–0.53)	**< 0.001**
DCP, ≤ 400 vs. >400, mAU/ml	-0.573	0.334	0.56 (0.29–1.09)	0.087	-0.399	0.275	0.67 (0.39–1.15)	0.147
HBV-DNA, ≤ 1000 vs. >1000, copies/ml	-1.064	0.372	0.35 (0.17–0.72)	**0.004**	-1.042	0.314	0.35 (0.19–0.65)	**0.001**
Tumor diameter, ≤ 10 vs. >10, cm	-0.490	0.367	0.61 (0.30–1.26)	0.181	-0.202	0.293	0.82 (0.46–1.45)	0.489
Tumor number, single vs. multiple	-0.528	0.605	0.59 (0.18–1.93)	0.383	-0.338	0.524	0.71 (0.26–1.99)	0.519
Extrahepatic metastasis, yes vs. no	1.122	0.611	3.07 (0.93–10.17)	0.066	0.817	0.598	2.26 (0.70–7.31)	0.172
Macrovascular invasion, yes vs. no	0.669	0.340	1.95 (1.00–3.80)	0.050	0.462	0.278	1.59 (0.92–2.74)	0.097

Notes: T2DM, type 2 diabetes mellitus; ECOG PS, Eastern Cooperative Oncology Group performance status; PLT, platelets; PT, prothrombin time; Scr, serum creatinine; TBIL, total bilirubin; ALB, albumin; AFP, alpha-fetoprotein; NLR, neutrophil-to-lymphocyte ratio; DCP, des-γ-carboxy-prothrombin

On multivariable Cox regression analyses, AFP (HR 0.48, 95% CI 0.24–0.97, *P* = 0.039) and NLR (HR 0.11, 95% CI 0.03–0.37, *P* < 0.001) remained as independent predictors of OS. NLR (HR 0.27, 95% CI 0.14–0.54, *P* < 0.001) remained as an independent prognostic factor of PFS (Table 3).

**Table 3:** Multivariable Cox regression analyses of prognostic factors for overall survival and progression-free survival

	Overall survival				Progression-free survival			
	β	SE	HR (95% CI)	*P* value	β	SE	HR (95% CI)	*P* value
AFP, ≤ 400 vs. >400 ng/ml	-0.730	0.354	0.48 (0.24–0.97)	**0.039**	-0.603	0.328	0.61 (0.36–1.05)	0.060
NLR, ≤ 2.77 vs. >2.77	-2.197	0.609	0.11 (0.03–0.37)	**<0.001**	-1.316	0.352	0.27 (0.14–0.54)	**<0.001**
HBV-DNA, ≤ 1000 vs. >1000 copies/ml	-0.495	0.356	0.55 (0.27–1.10)	0.091	-0.401	0.277	0.67 (0.39–1.15)	0.148

Notes: AFP, alpha-fetoprotein; NLR, neutrophil-to-lymphocyte ratio

### Establishment of a score to predict survival, tumor response and disease control for HCC Patients undergoing immunotherapy

Next, we aimed to establish an objective, simple, laboratory indicator-based score to predict long-term survival, tumor response and disease control in HCC patients who were treated with immunotherapy. The regression coefficients (β, B-values) of multivariate Cox regression analyses of OS were multiplied by a factor of 1.37, and the maximum number of integers was determined to calculate the immunotherapy score. We assigned 1 point for an AFP level > 400 ng/mL and 3 points for an NLR value > 2.77. Hence, an individual patient could get either 0 (both AFP ≤ 400 ng/mL and NLR ≤ 2.77), 1 (AFP > 400 ng/mL and NLR ≤ 2.77), 3 (AFP ≤ 400 ng/mL and NLR > 2.77), or 4 (both AFP > 400 ng/mL and NLR > 2.77) points. HCC patients with 0 point were classified as the low-risk group. HCC patients with 1 point or 3 points were categorized as the intermediate-risk group. HCC patients with 4 points were classified as the high-risk group.

**Fig. 4:**
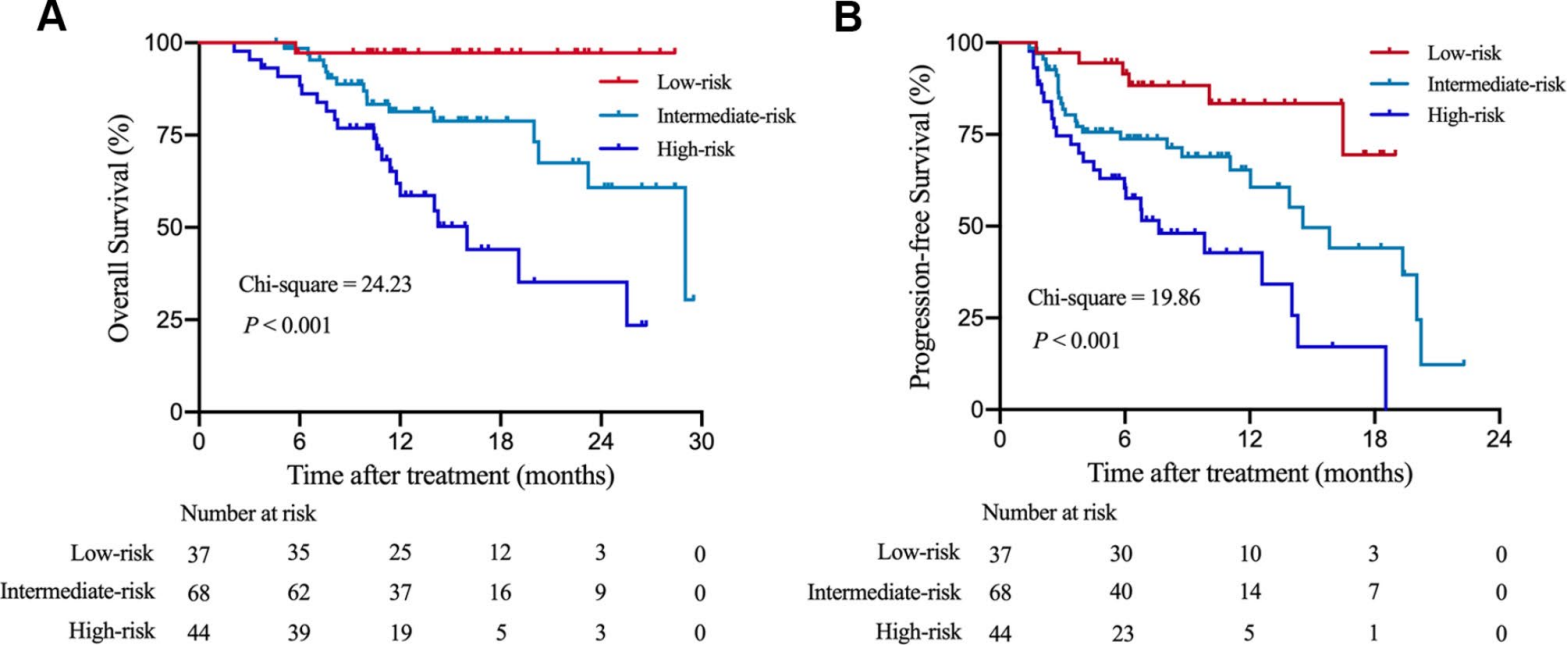
Kaplan-Meier survival curves of the low- (0 point, n = 37), intermediate- (1 point or 3 points, n = 68), and high-risk (4 points, n = 44) groups according to the HCC immunotherapy score in the internal training cohort. (**A**) the prognostic significance of the three subgroups for overall survival (OS); (**B**) the prognostic significance of the three subgroups for progression-free survival (PFS).

In the internal training cohort, the median OS of the low-risk group was not reached. The median OS of the intermediate-risk group and high-risk group were 29.0 (95% CI 20.8–37.3) months and 16.0 (95% CI 10.8–21.2) months, respectively (*P* < 0.001) (Fig. 4A). The median PFS of the low-risk group was not reached. The median PFS of the intermediate-risk group and high-risk group were 14.6 (95% CI 11.3–17.8) months and 7.6 (95% CI 3.6–11.7) months, respectively (*P* < 0.001) (Fig. 4B).

Then, the prognostic performance of the immunotherapy scoring system was assessed in the independent external validation cohort. As shown in **Figure **S2, the median OS of the low-risk group was not reached. The median OS of the intermediate-risk group and high-risk group were 29.0 (95% CI 20.4–37.6) months and 16.0 (95% CI 11.1–20.9) months, respectively (*P* < 0.001) (**Figure **S2A). The median PFS of the low-risk group was not reached. The median PFS of the intermediate-risk group and high-risk group were 13.9 (95% CI 9.6–18.2) months and 7.6 (95% CI 3.2–12.0) months, respectively (*P* = 0.002) (**Figure **S2B).

In addition, the clinical utility of this score to estimate tumor response and disease control to immunotherapy was evaluated. As shown in **Table **S4 and **Table **S5, The ORR and DCR in the training cohort were 51.7% and 74.5%, respectively. The ORR and DCR in the validation cohort were 53.0% and 76.0%, respectively. The ORR was highest in the low-risk group, followed by the intermediate group and the high-risk group both in the training and validation cohorts (**Table **S4, *P* < 0.001 and *P* = 0.003). Also, the DCR was best in the low-risk group, followed by the intermediate group and the high-risk group both in the training and validation cohorts (**Table **S5, *P* = 0.007 and *P* = 0.013).

All these results demonstrated that this score had a good discriminatory power in selecting patients who would gain survival benefit from immunotherapy.

## Discussion

It has been well established that both AFP and NLR are prognostic factors in HCC and have been incorporated into different prognostic models [19–23]. In the current study, we constructed a simple and practical score using pre-treatment serum AFP and NLR, which can predict the overall survival and progression-free survival in patients with unresectable HCC who received immunotherapy. Using this score, these patients were stratified into three distinct groups. Accordingly, in contrast to patients with AFP > 400 ng/mL and NLR > 2.77, patients who fulfilled none of these criteria had the best survival; patients who fulfilled only one criterion still had an improved survival outcome. Using this HCC immunotherapy score, tumor response and disease control rates were also stratified well among the three risk groups. HCC patients in the low-risk group had the best ORR and DCR, followed by the intermediate-risk and high-risk groups.

There is a good rationale to combine AFP and NLR to predict the treatment response and survival outcome of HCC patients receiving immunotherapy. AFP is a conventional diagnostic biomarker of HCC in clinical practice, and also a potential target for immunotherapy [24]. Emerging evidence suggests that AFP is closely associated with the prognosis of patients with HCC treated with immunotherapy. A clinical trial reported that AFP had important value in immunotherapy response prediction for HCC patients [25]. Sun et al. [26]. found that early reduction in AFP level could precisely predict the effectiveness of PD-1 inhibitor in HCC patients. Moreover, another study demonstrated that the dynamic changes of AFP level was able to accurately reflect the therapeutic response and predict prognosis in HCC patients receiving ICI-based treatment [13]. Consistent with their findings, our study showed that AFP less than 400 ng/mL was associated with significantly better OS (HR = 0.48, 95% CI 0.24–0.97, *P* = 0.039).

Chronic inflammation and evasion of immune surveillance are recognized as cancer hallmarks [27]. Neutrophil-to-lymphocyte ratio (NLR) is easily measurable with a ratio that can be simply calculated from a complete blood count. NLR has been proved to be associated with prognosis for patients with various cancers in diverse clinical settings [28–31]. It was also found to have a prognostic role in patients with different solid tumors undergoing ICIs [32–34]. Preoperative NLR may serve as a surrogate marker of the balance between pro-tumoral inflammatory status and anti-tumoral immune response. Neutrophilia hinders immunotherapy efficacy as it suppresses the immune system and is associated with high production of chemokines and cytokines, which contribute to tumor progression [35, 36]. Depleted lymphocyte is also associated with impaired antitumor immune responses [37]. In the present study, HCC patients with NLR ≤ 2.77 who received immunotherapy had significantly better PFS and OS (both *P*<0.001) than those with NLR > 2.77, which reflected the potential utility of NLR to predict survival in HCC patients undergoing ICIs.

Several limitations to this study should be acknowledged. First, as this is a non-randomized retrospective study, unrecognized selection biases may confound the findings. Second, this score is constructed based on a single-center training cohort and only verified in an external validation cohort. The accuracy and predictive power of this score should be further verified in prospective internal and more independent validation cohorts. Third, most of the patients enrolled in this study had a background of hepatitis B virus infection. Therefore, whether this score can be extrapolated to patients with other etiologies needs further studies. Last, patients of this study received various kinds of anti-PD-1/PD-L1 dominant treatments and some patients underwent combined therapy, which may bring inconformity in treatment course. However, the results could better reflect the real-world situation.

In conclusion, we established a score combining baseline AFP and NLR to predict survival outcomes and treatment response of patients receiving anti-PD-1/PD-L1 dominant treatments for unresectable HCC. As this score is based on two ubiquitously available laboratory values, it is objective and practical. Moreover, this score could assist in the selection of patients who are most likely to benefit from immunotherapy and guide clinical treatment decision-making. Nervertheless, this score warrants further prospective validation in a large clinical study.

## Electronic supplementary material

Below is the link to the electronic supplementary material.

Supplementary Material 1

Supplementary Material 2

TABLE S1 Category and dosage of PD-1/PD-L1 inhibitors used in the study for the internal training and external validation cohorts

TABLE S2 Comparison of baseline patient demographics and disease characteristics between the low and high AFP groups

TABLE S3 Comparison of baseline patient demographics and disease characteristics between the low and high NLR groups

TABLE S4 Comparison of tumor response in different risk groups using the immunotherapy score

TABLE S5 Comparison of disease control in different risk groups using the immunotherapy score

## Data Availability

The data that support the findings of this study are available from the corresponding author upon reasonable request.
