# Supplementary figures and images for: Combination of alpha-fetoprotein and neutrophil-to-lymphocyte ratio to predict treatment response and survival outcomes of patients with unresectable hepatocellular carcinoma treated with immune checkpoint inhibitors
Source: BMC Cancer. 2023 Jun 15;23:547. doi: 10.1186/s12885-023-11003-0 (PMC10268526; doi:10.1186/s12885-023-11003-0)

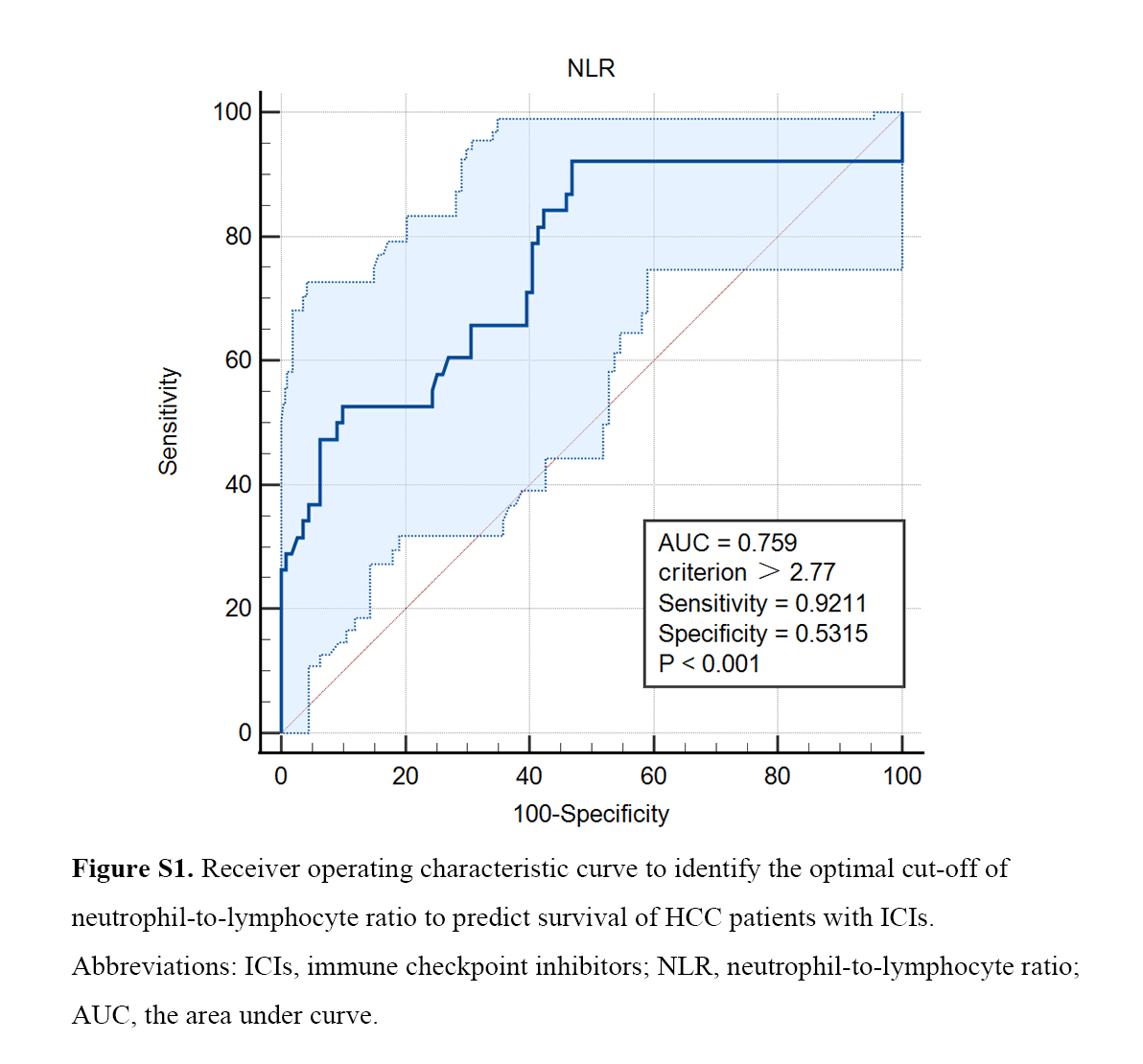

Supplement: Supplementary file 1 — Supplementary Material 1 [file 12885_2023_11003_MOESM1_ESM.png]

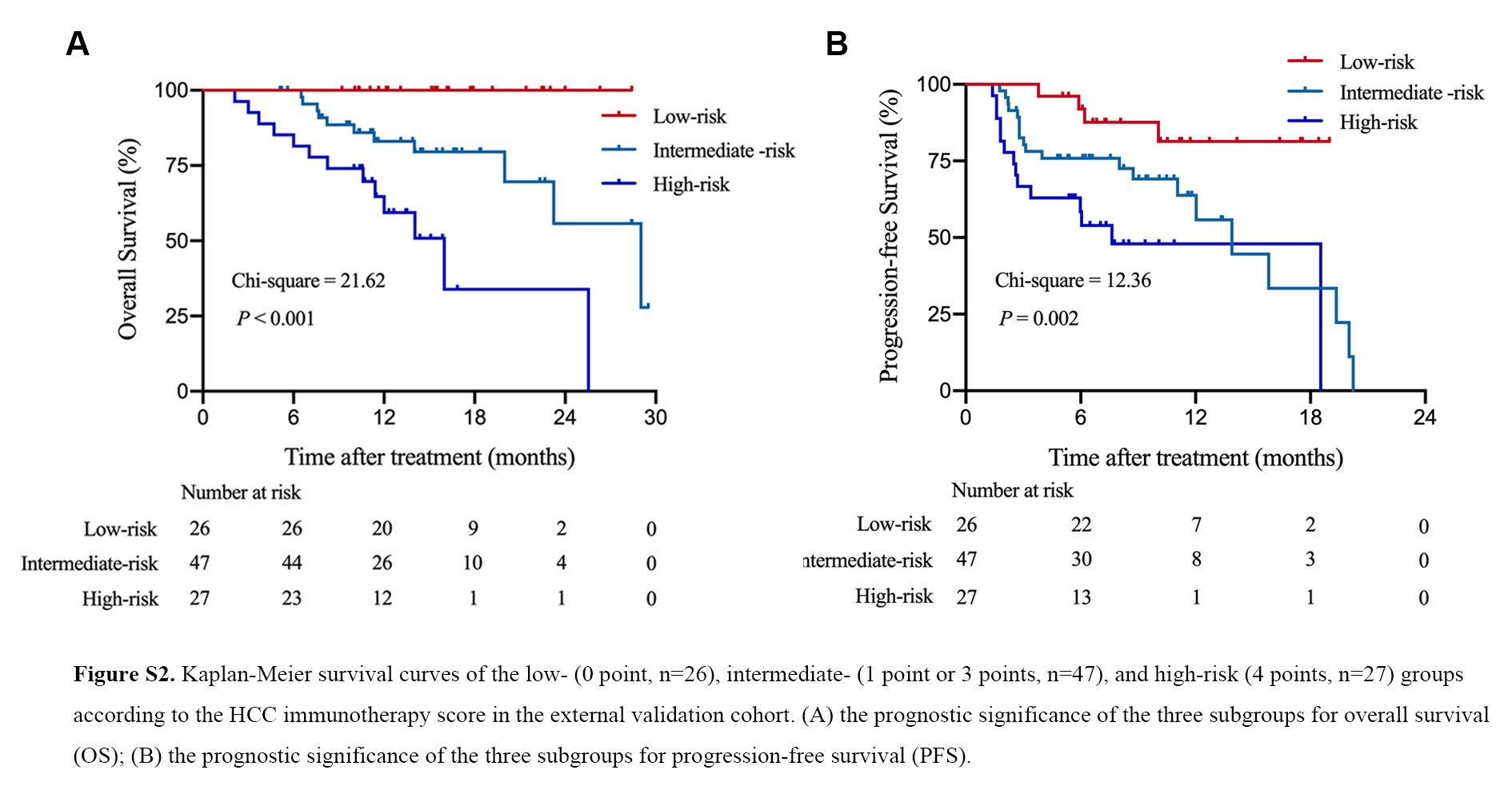

Supplement: Supplementary file 2 — Supplementary Material 2 [file 12885_2023_11003_MOESM2_ESM.png]
