# Supplementary material for: Combination of alpha-fetoprotein and neutrophil-to-lymphocyte ratio to predict treatment response and survival outcomes of patients with unresectable hepatocellular carcinoma treated with immune checkpoint inhibitors
Source: BMC Cancer. 2023 Jun 15;23:547. doi: 10.1186/s12885-023-11003-0 (PMC10268526; doi:10.1186/s12885-023-11003-0)
Supplement: Supplementary file 3 — TABLE S1 Category and dosage of PD-1/PD-L1 inhibitors used in the study for the internal training and external validation cohorts [file 12885_2023_11003_MOESM3_ESM.docx]

**TABLE S1 Category and dosage of PD-1/PD-L1 inhibitors used in the study for the internal training and external validation cohorts**

| **Patient cohort** | | Internal training cohort | External validation cohort |
| --- | --- | --- | --- |
| Category | Dose (mg) | N=149 No. (%) | N=100 No. (%) |
| Camrelizumab (Suzhou, shengdiya pharmaceutical Co. Ltd) | 200 | 16 (10.7) | 14 (14.0) |
| Sintilimab (Suzhou, xinda pharmaceutical Co. Ltd) | 200 | 44 (29.5) | 26 (26.0) |
| Toripalimab (Suzhou, hezhong pharmaceutical Co. Ltd) | 240 | 85 (57.0) | 58 (58.0) |
| Tislelizumab (Shanghai, baijishenzhou pharmaceutical Co. Ltd) | 200 | 3 (2.0) | 2 (2.0) |
| Atezolizumab (Roche Pharmaceuticals Co. Ltd) | 1200 | 1 (0.7) | 0 (0) |
