# Supplementary material for: Combination of alpha-fetoprotein and neutrophil-to-lymphocyte ratio to predict treatment response and survival outcomes of patients with unresectable hepatocellular carcinoma treated with immune checkpoint inhibitors
Source: BMC Cancer. 2023 Jun 15;23:547. doi: 10.1186/s12885-023-11003-0 (PMC10268526; doi:10.1186/s12885-023-11003-0)
Supplement: Supplementary file 4 — TABLE S2 Comparison of baseline patient demographics and disease characteristics between the low and high AFP groups [file 12885_2023_11003_MOESM4_ESM.docx]

**TABLE S2 Comparison of baseline patient demographics and disease characteristics between the low and high AFP groups**

| **Characteristics** | **Internal training**  **cohort**  **N=149 (%)** | | | **External validation**  **cohort**  **N=100 (%)** | | |
| --- | --- | --- | --- | --- | --- | --- |
|  | AFP ≤ 400  N=76 | AFP > 400  N=73 | *P* | AFP ≤ 400  N=52 | AFP > 400  N=48 | *P* |
| Age, years | 58 (52–63) | 53 (46–60) | **0.004** | 58 (52–62) | 53 (40–63) | **0.032** |
| Age, < 65/≥ 65 years | 60/16 | 63/10 | 0.237 | 46/6 | 40/8 | 0.460 |
| Sex, male/female | 64/12 | 61/12 | 0.914 | 43/9 | 36/12 | 0.345 |
| Etiology, HBV/Non-HBV | 60/16 | 65/8 | 0.094 | 43/9 | 43/5 | 0.321 |
| Cardiovascular diseases, yes/no | 45/31 | 50/23 | 0.239 | 31/21 | 28/20 | 0.896 |
| T2DM, yes/no | 21/55 | 21/52 | 0.878 | 15/37 | 10/38 | 0.355 |
| Antiviral treatment, yes/no | 30/46 | 42/31 | **0.027** | 22/30 | 24/24 | 0.441 |
| Child-Pugh class, A/B | 69/7 | 60/13 | 0.124 | 45/7 | 39/9 | 0.471 |
| ECOG PS, 0–1/2 | 68/8 | 73/0 | **0.006** | 50/2 | 48/0 | 0.496 |
| BCLC, B/C | 57/19 | 47/26 | 0.158 | 39/13 | 31/17 | 0.256 |
| PLT, ×10^9^/L | 160.0 ± 53.2 | 155.0 ± 65.1 | 0.612 | 159.4 ± 58.1 | 156.1 ± 61.9 | 0.786 |
| PT, s | 12.2 (11.4–12.9) | 12.3 (11.9–13.2) | **0.036** | 12.4 (11.2–12.9) | 12.6 (12.2–13.3) | **0.046** |
| Scr, umol/L | 68 (62–82) | 64 (57–80) | 0.173 | 64 (57–68) | 58 (55–64) | **0.008** |
| Blood glucose, mmol/L | 5.64 (4.83–7.61) | 4.98 (4.43–5.90) | **0.001** | 5.67 (4.85–8.11) | 5.00 (4.49–5.90) | **0.006** |
| TBIL, umol/L | 16.0 (10.9–21.9) | 18.6 (12.7–23.5) | 0.109 | 15.7 (10.9–21.9) | 17.8 (12.5–24.0) | 0.131 |
| ALB, g/L | 41.0 (36.1–42.4) | 37.9 (35.5–41.9) | 0.123 | 40.3 (35.3–42.4) | 38.0 (35.4–42.0) | 0.417 |
| ALBI score | -2.68 (-2.95–-2.30) | -2.36 (-2.83–-2.08) | **0.005** | -2.66 (-2.83–-2.24) | -2.39 (-2.80–-2.01) | 0.091 |
| ALBI grade, I/II/III | 44/29/3 | 23/49/1 | **0.002** | 28/21/3 | 14/33/1 | **0.017** |
| NLR, ≤2.77/ >2.77 | 35/41 | 27/46 | 0.262 | 25/27 | 20/28 | 0.520 |
| DCP, ≤400/ >400 mAU/ml | 37/39 | 10/63 | **<0.001** | 28/24 | 6/42 | **<0.001** |
| HBV-DNA, ≤1000/ >1000 copies/ml | 67/9 | 57/16 | 0.100 | 47/5 | 37/11 | 0.070 |
| Macrovascular invasion, yes/no | 19/57 | 26/47 | 0.158 | 13/39 | 17/31 | 0.256 |
| Extrahepatic metastasis, yes/no | 1/75 | 6/67 | 0.060 | 0/52 | 6/42 | **0.010** |
| Tumor number, single/multiple | 6/70 | 6/67 | 0.942 | 5/47 | 3/45 | 0.717 |
| Largest tumor size, cm | 9.0 (6.5–13.0) | 10.5 (6.9–13.3) | 0.118 | 9.0 (6.2–13.0) | 10.9 (7.7–13.5) | **0.022** |
| Combined treatment besides ICIs, TACE/TKI*/PMCT/RT | 46/30/8/1 | 85/60/4/2 | 0.156 | 24/15/4/1 | 68/50/2/3 | 0.145 |

Notes: values are presented as mean ± SD, median (interquartile range), or numbers.

*TKI include Sorafenib, Lenvatinib, Regorafenib and Apatinib.

AFP, alpha-fetoprotein; HBV, hepatitis B virus; T2DM, type 2 diabetes mellitus; ECOG PS, Eastern Cooperative Oncology Group performance status; BCLC, Barcelona Clinic Liver Cancer; PLT, platelets; PT, prothrombin time; Scr, serum creatinine; TBIL, total bilirubin; ALB, albumin; ALBI, albumin-bilirubin; NLR, neutrophil-to-lymphocyte ratio; DCP, des-γ-carboxy-prothrombin; ICIs, immune checkpoint inhibitors; TACE, transcatheter arterial chemoembolization; TKI, tyrosine kinase inhibitor; PMCT, percutaneous microwave coagulation therapy; RT, radiotherapy.

|  | AFP ≤ 400 | AFP > 400 | *P* |
| --- | --- | --- | --- |
| Age | 57.1 ± 9.3 | 52.0 ± 11.1 | 0.053 |
| Sex, male/female | 67/13 | 58/11 | 0.959 |
| Child-Pugh stage, A/B | 72/8 | 57/12 | 0.187 |
| ECOG PS, 0-1/2 | 72/8 | 69/0 | 0.088 |
| BCLC, B/C | 60/20 | 44/25 | 0.136 |
| PLT, ×10^9^/L | 157.7 ± 53.4 | 157.3 ± 65.5 | 0.967 |
| PT, s | 12.3 ± 1.0 | 12.7 ± 1.1 | 0.111 |
| Scr, umol/L | 70.4 ± 15.1 | 68.1 ± 15.4 | 0.359 |
| TBIL, umol/L | 17.5 ± 8.3 | 21.0 ± 10.9 | 0.069 |
| ALB, g/L | 40.7 ± 7.2 | 38.8 ± 4.3 | 0.059 |
| NLR, ≤2.77/>2.77 | 37/43 | 25/44 | 0.216 |
| Macrovascular invasion, yes/no | 20/60 | 25/44 | 0.136 |
| Extrahepatic metastasis, yes/no | 1/79 | 6/63 | 0.080 |
| Tumor number, single/multiple | 6/74 | 6/63 | 0.789 |
| Largest tumor size, cm | 9.3 ± 3.8 | 10.8 ± 4.4 | 0.050 |
| Previous treatment, yes/no | 53/27 | 48/21 | 0.666 |

Notes: values are presented as mean ± SD or numbers.

AFP, alpha-fetoprotein; ECOG PS, Eastern Cooperative Oncology Group performance status; BCLC, Barcelona Clinic Liver Cancer; PLT, platelets; PT, prothrombin time; Scr, serum creatinine; TBIL, total bilirubin; ALB, albumin; NLR, neutrophil-to-lymphocyte ratio.
