# Supplementary material for: Combination of alpha-fetoprotein and neutrophil-to-lymphocyte ratio to predict treatment response and survival outcomes of patients with unresectable hepatocellular carcinoma treated with immune checkpoint inhibitors
Source: BMC Cancer. 2023 Jun 15;23:547. doi: 10.1186/s12885-023-11003-0 (PMC10268526; doi:10.1186/s12885-023-11003-0)
Supplement: Supplementary file 5 — TABLE S3 Comparison of baseline patient demographics and disease characteristics between the low and high NLR groups [file 12885_2023_11003_MOESM5_ESM.docx]

**TABLE S3 Comparison of baseline patient demographics and disease characteristics between the low and high NLR groups**

| **Characteristics** | **Internal training**  **cohort**  **N=149 (%)** | | | **External validation**  **cohort**  **N=100 (%)** | | |
| --- | --- | --- | --- | --- | --- | --- |
|  | NLR ≤ 2.77  N=62 | NLR > 2.77  N=87 | *P* | NLR ≤ 2.77  N=45 | NLR > 2.77  N=55 | *P* |
| Age, years | 56 (50–61) | 56 (47–61) | 0.452 | 56 (48–61) | 57 (47–63) | 0.945 |
| Age, < 65/≥ 65 years | 47/15 | 76/11 | 0.067 | 38/7 | 48/7 | 0.685 |
| Sex, male/female | 51/11 | 74/13 | 0.647 | 37/8 | 42/13 | 0.474 |
| Etiology, HBV/Non-HBV | 52/10 | 73/14 | 0.995 | 41/4 | 45/10 | 0.183 |
| Cardiovascular diseases, yes/no | 38/24 | 57/30 | 0.597 | 28/17 | 31/24 | 0.553 |
| T2DM, yes/no | 16/46 | 26/61 | 0.585 | 10/35 | 15/40 | 0.562 |
| Antiviral treatment, yes/no | 29/33 | 43/44 | 0.750 | 19/26 | 27/28 | 0.493 |
| Child-Pugh class, A/B | 54/8 | 75/12 | 0.875 | 38/7 | 46/9 | 0.913 |
| ECOG PS, 0–1/2 | 56/6 | 85/2 | 0.067 | 44/1 | 54/1 | 1.000 |
| BCLC, B/C | 42/20 | 62/25 | 0.644 | 29/16 | 41/14 | 0.273 |
| PLT, ×10^9^/L | 157.4 ± 55.2 | 157.6 ± 62.1 | 0.984 | 162.8 ± 59.9 | 153.8 ± 59.7 | 0.456 |
| PT, s | 12.4 (12.0–13.4) | 12.2 (11.7–12.9) | 0.051 | 12.7 (12.1–13.5) | 12.3 (11.8–12.9) | 0.072 |
| Scr, umol/L | 66 (57–76) | 68 (59–81) | 0.304 | 60 (55–67) | 62 (56–67) | 0.675 |
| Blood glucose, mmol/L | 5.42 (4.81–6.35) | 5.03 (4.53–6.15) | 0.286 | 5.18 (4.81–6.45) | 5.15 (4.54–5.94) | 0.640 |
| TBIL, umol/L | 17.6 (12.5–23.4) | 16.4 (11.9–22.5) | 0.673 | 15.6 (12.0–24.8) | 16.3 (12.0–22.6) | 0.956 |
| ALB, g/L | 37.9 (35.5–41.9) | 40.3 (35.9–42.6) | 0.113 | 37.9 (35.2–41.9) | 40.2 (35.5–42.7) | 0.242 |
| ALBI score | -2.44 (-2.82–-2.11) | -2.61 (-2.90–-2.11) | 0.278 | -2.42 (-2.71–-2.08) | -2.52 (-2.86–-2.08) | 0.356 |
| ALBI grade, I/II/III | 24/38/0 | 43/40/4 | 0.067 | 16/28/1 | 26/26/3 | 0.289 |
| AFP, ≤400/ >400 ng/ml | 35/27 | 41/46 | 0.262 | 25/20 | 27/28 | 0.520 |
| DCP, ≤400/ >400 mAU/ml | 18/44 | 29/58 | 0.578 | 14/31 | 20/35 | 0.581 |
| HBV-DNA, ≤1000/ >1000 copies/ml | 57/5 | 67/20 | **0.016** | 42/3 | 42/13 | **0.021** |
| Macrovascular invasion, yes/no | 20/42 | 25/62 | 0.644 | 16/29 | 14/41 | 0.273 |
| Extrahepatic metastasis, yes/no | 4/58 | 3/84 | 0.451 | 3/42 | 3/52 | 0.800 |
| Tumor number, single/multiple | 4/58 | 8/79 | 0.544 | 2/43 | 6/49 | 0.289 |
| Largest tumor size, cm | 9.0 (6.2–12.4) | 10.1 (7.3–13.2) | 0.084 | 9.5 (6.2–12.8) | 10.3 (7.4–13.3) | 0.786 |
| Combined treatment besides PD-1 inhibitors, TACE/TKI*/PMCT/RT | 41/24/7/1 | 90/66/5/2 | 0.172 | 28/20/5/2 | 64/45/1/2 | 0.107 |

Notes: values are presented as mean ± SD, median (interquartile range), or numbers.

*TKI include Sorafenib, Lenvatinib, Regorafenib and Apatinib.

NLR, neutrophil-to-lymphocyte ratio; HBV, hepatitis B virus; T2DM, type 2 diabetes mellitus; ECOG PS, Eastern Cooperative Oncology Group performance status; BCLC, Barcelona Clinic Liver Cancer; PLT, platelets; PT, prothrombin time; Scr, serum creatinine; TBIL, total bilirubin; ALB, albumin; ALBI, albumin-bilirubin; AFP, alpha-fetoprotein; DCP, des-γ-carboxy-prothrombin; ICIs, immune checkpoint inhibitors; TACE, transcatheter arterial chemoembolization; TKI, tyrosine kinase inhibitor; PMCT, percutaneous microwave coagulation therapy; RT, radiotherapy.
