# Supplementary material for: Combination of alpha-fetoprotein and neutrophil-to-lymphocyte ratio to predict treatment response and survival outcomes of patients with unresectable hepatocellular carcinoma treated with immune checkpoint inhibitors
Source: BMC Cancer. 2023 Jun 15;23:547. doi: 10.1186/s12885-023-11003-0 (PMC10268526; doi:10.1186/s12885-023-11003-0)
Supplement: Supplementary file 6 — TABLE S4 Comparison of tumor response in different risk groups using the immunotherapy score [file 12885_2023_11003_MOESM6_ESM.docx]

**TABLE S4 Comparison of tumor response in different risk groups using the immunotherapy score**

|  | *Internal training cohort* | | | | *External validation cohort* | | | |
| --- | --- | --- | --- | --- | --- | --- | --- | --- |
| Tumor response | Low-risk group  n (%) | Intermediate-risk group  n (%) | High-risk group  n (%) | *P* | Low-risk group  n (%) | Intermediate-risk group  n (%) | High-risk group  n (%) | *P* |
| Response | 29 (78.4) | 37 (54.4) | 11 (25.0) | < 0.001 | 20 (76.9) | 25 (53.2) | 8 (29.6) | 0.003 |
| Non-response | 8 (21.6) | 31 (45.6) | 33 (75.0) |  | 6 (23.1) | 22 (46.8) | 19 (70.4) |  |
