# Supplementary material for: Combination of alpha-fetoprotein and neutrophil-to-lymphocyte ratio to predict treatment response and survival outcomes of patients with unresectable hepatocellular carcinoma treated with immune checkpoint inhibitors
Source: BMC Cancer. 2023 Jun 15;23:547. doi: 10.1186/s12885-023-11003-0 (PMC10268526; doi:10.1186/s12885-023-11003-0)
Supplement: Supplementary file 7 — TABLE S5 Comparison of disease control in different risk groups using the immunotherapy score [file 12885_2023_11003_MOESM7_ESM.docx]

**TABLE S5 Comparison of disease control in different risk groups using the immunotherapy score**

|  | *Internal training cohort* | | | | *External validation cohort* | | | |
| --- | --- | --- | --- | --- | --- | --- | --- | --- |
| Disease control | Low-risk group  n (%) | Intermediate-risk group  n (%) | High-risk group  n (%) | *P* | Low-risk group  n (%) | Intermediate-risk group  n (%) | High-risk group  n (%) | *P* |
| Stable disease | 34 (91.9) | 50 (73.5) | 27 (61.4) | 0.007 | 25 (96.2) | 34 (72.3) | 17 (63.0) | 0.013 |
| Disease progression | 3 (8.1) | 18 (26.5) | 17 (38.6) |  | 1 (3.8) | 13 (27.7) | 10 (37.0) |  |
